# High-Performance Wearable Strain Sensor Based on MXene@Cotton Fabric with Network Structure

**DOI:** 10.3390/nano11040889

**Published:** 2021-03-31

**Authors:** Lu Liu, Libo Wang, Xuqing Liu, Wenfeng Yuan, Mengmeng Yuan, Qixun Xia, Qianku Hu, Aiguo Zhou

**Affiliations:** 1 Henan Key Laboratory of Materials on Deep-Earth Engineering, School of Materials Science and Engineering, Henan Polytechnic University, Jiaozuo 454000, China; liulu0804@163.com (L.L.); ywf3507@163.com (W.Y.); YMM19980317@163.com (M.Y.); xqx@hpu.edu.cn (Q.X.); hqk@hpu.edu.cn (Q.H.); 2 Department of Materials, The University of Manchester, Oxford Road, Manchester M13 9PL, UK; xuqing.liu@manchester.ac.uk

**Keywords:** MXene, cotton fabric, strain sensor, flexible

## Abstract

Flexible and comfortable wearable electronics are as a second skin for humans as they can collect the physiology of humans and show great application in health and fitness monitoring. MXene Ti_3_C_2_T_x_ have been used in flexible electronic devices for their unique properties such as high conductivity, excellent mechanical performance, flexibility, and good hydrophilicity, but less research has focused on MXene-based cotton fabric strain sensors. In this work, a high-performance wearable strain sensor composed of two-dimensional (2D) MXene d-Ti_3_C_2_T_x_ nanomaterials and cotton fabric is reported. Cotton fabrics were selected as substrate as they are comfortable textiles. As the active material in the sensor, MXene d-Ti_3_C_2_T_x_ exhibited an excellent conductivity and hydrophilicity and adhered well to the fabric fibers by electrostatic adsorption. The gauge factor of the MXene@cotton fabric strain sensor reached up to 4.11 within the strain range of 15%. Meanwhile, the sensor possessed high durability (>500 cycles) and a low strain detection limit of 0.3%. Finally, the encapsulated strain sensor was used to detect subtle or large body movements and exhibited a rapid response. This study shows that the MXene@cotton fabric strain sensor reported here have great potential for use in flexible, comfortable, and wearable devices for health monitoring and motion detection.

## 1. Introduction

Compared to traditional electronic devices, flexible electronic devices have good flexibility, ductility, and can be easily bent, twisted, or folded [1]. Therefore, these flexible devices can meet the deformation requirements of equipment under different working conditions and have broad applications in flexible electronic displays [2], thin-film solar cells [3], flexible sensors [4], and wearable devices [5]. Among these, flexible wearable electronic devices have attracted considerable attention due to their enormous potential applications in personal health monitoring, biomedical research, and artificial intelligence [6,7,8]. Moreover, due to their simple structure and operating principles, superior sensing properties to various types of deformations, and good expansibility, resistance-type flexible stress/strain sensors have very broad application prospects in many fields [9,10]. Wearable stress/strain sensors are in great demand with the rapid development of intelligent electronic devices. However, obtaining high-performance pressure sensors that simultaneously have a high sensitivity, wide response range, and low detection limit is still a considerable challenge. Thus, considerable research into the processing, structures, and materials of different sensors has been conducted in recent years to meet the demands for intelligent wearable devices and the manufacture of wearable electronic sensors with high sensitivity, high resolution, and fast responses.

The choice of the underlying comfortable and flexible substrate has an important impact on the ultimate performance of the flexible stress/strain sensor. According to the material dimensions, flexible substrates can be divided into three types: One-dimensional fibers or yarn [11,12], two-dimensional films [13] and fabrics [14], and three-dimensional sponges/foams [15], gels [16] and other architectures [17]. Fabric-based flexible stress/strain sensors have obvious advantages in flexible electronic devices as they can withstand complicated deformations, such as bending, stretching, and twisting, and are made from simple and low-cost materials [18,19]. Compared with other intelligent electronic devices such as membranes and foams, textile-based flexible mechanical sensors also can achieve a seamless connection with clothing, home textiles and other fabric products, and realize an integrated design. On the other hand, the active layer and the morphologies and microstructures of the sensing materials have also been widely recognized as important factors for improving the sensitivity of sensors. Carbon nanotubes [20,21], graphene [22], metal nanowires [23], and other nanomaterials often are used in the active layer due to their excellent properties. However, it is still difficult and complicated to fabricate highly sensitive fabric-based flexible sensors due to low signal responses. Therefore, more research efforts should focus on developing of sensing materials and the fabrication processes of sensors.

2D materials are very suitable for use as flexible piezoresistive sensor materials that can meet the performance requirements of high sensitivity over a wide sensing range in flexible strain/stress sensors due to their large specific surface area, strong mechanical properties, and adjustable electrical properties [24]. As a new type of 2D materials, early transition metal carbides and/or nitrides, MXene, have attracted great attention [25,26,27]. Due to its unique 2D structure, MXene has displayed excellent performance in many fields, such as catalysis [28], energy storage [29,30], electromagnetic shielding [31], reinforced materials [32,33], and other fields [34,35,36]. For example, Guo et al. [37] fabricated a highly sensitive, flexible, and degradable pressure sensor by sandwiching porous MXene-impregnated tissue paper, which exhibited high sensitivity with a low detection limit (10.2 Pa), broad range (up to 30 kPa), fast response (11 ms), low power consumption (10–8 W), great reproducibility over 10,000 cycles, and excellent degradability. Li et al. [38] prepared a 2D MXene/(0D-1D) silver nanocomposite-based strain sensor, which when incorporated into fabric, could act as an electrothermal device. Their composite yarn strain sensor had a remarkably high strain sensitivity, effectively monitoring both the large and small deformations of various parts of the human body. Li et al. [39] proposed a flexible piezoresistive pressure sensor based on MXene-textiles prepared by a facile dip-coating process. The resulting pressure sensor exhibited high sensitivity with a rapid response time of 26 ms and excellent cycling stability. Liu et al. [40] fabricated a MXene-coated cotton fabric pressure sensor that showed a high gauge factor (7.67 kPa^−1^), a rapid response and relaxation speed (<35 ms), excellent stability (>2000 cycles), and good durability after washing. However, the theoretical research of fabric sensors and industrially applicable research still needs to be improved in many ways and studied in more depth. 

Herein, a highly sensitive MXene-based flexible strain sensor was fabricated. An immersion method was employed to uniformly deposit a layer of Ti_3_C_2_T_x_ MXene nanosheets onto comfortable cotton fabric through electrostatic interactions. The applied strain was increased on the MXene-based sensor, and the network structure of the fabric effectively increased the contact area between the conductive MXene channels, which led to an improved sensing performance of the strain sensor. The obtained strain sensor exhibited outstanding sensitivity (GF = 4.11), a subtle strain detection limit (0.3%), and excellent stability over 500 cycles. 

## 2. Experimental

### 2.1. Preparation of MXene Ti_3_C_2_T_X_ and Exfoliated d-Ti_3_C_2_T_X_ Nanosheets

MAX phase Ti_3_AlC_2_, the precursor of MXene Ti_3_C_2_T_x_, was synthetized by pressless sintering technology and passed 500 mesh sieves in the previous report [41]. MXene Ti_3_C_2_T_x_ was synthesized by selectively exfoliated “Al” atoms from Ti_3_AlC_2_ in a hydrochloric acid solution of sodium fluoride [42]. Subsequently, Ti_3_C_2_T_x_ powders were added to dimethyl sulphoxide (DMSO) and magnetically stirred at room temperature for 18 h. Then, the mixture was centrifuged at 8000 rpm for 10 min to obtain the precipitate, which was redispersed in 500 mL DI water, and ultrasonicated for 6 h under Ar flow. After that, the suspension was centrifuged at 3500 rpm for 1 h. The supernatant was collected to obtain the delaminated MXene suspension and labelled as “d-Ti_3_C_2_T_x_”.

### 2.2. Fabrication of MXene@Cotton Fabric Pressure/Strain Sensors

Typically, cotton fabric was wrapped by MXene sheets by immersion process. First, the cotton fabric was cut into rectangular shape with proper dimensions, which were washed with deionized water and ethanol several times to remove the impurities, and dried in a vacuum oven. Then, cotton fabric was immersed in the polyethyleneimine (PEI) aqueous solution (0.5 mg mL^−1^) for 24 h. The PEI was adsorbed on the surface of the cotton fabric, and forming positively charged cotton fabric@PEI, and drying in vacuum at 60 °C for 2 h. Second, cotton fabric@PEI was immersed into the delaminated d-Ti_3_C_2_T_x_ dispersions for 1 h. Due to the strong electrostatic interaction between MXene sheets and PEI, the negatively charged d-Ti_3_C_2_T_x_ nanosheets were wrapped on the cotton fabric. Finally, the samples were dried in a vacuum at 50 °C for 5 h, and MXene@Cotton Fabric strain sensors were obtained, labelled as a “MCF” strain sensor.

### 2.3. Characterization

The morphologies and microstructures of the MXene, Cotton Fabric, and MCF strain sensors were observed by field emission scanning electron microscope (FESEM) (S4800, Hitachi, Tokyo, Japan) with integrated energy-dispersive X-ray spectroscopy (EDS) for element analysis. The crystal structure of the samples was determined by X-ray diffractometer (XRD) (D8 Advance, Bruker, Billerica, MA, USA) equipment with Cu Kα1 radiation (λ = 0.154 nm) with a scanning rate of 15°/min and a step size of 0.02° from 5° to 80°. Thickness of d-Ti_3_C_2_T_x_ was characterized by the atomic force microscopy (AFM) (FM-Nanoview6800, Suzhou, China,). The FTIR spectra were recorded on a FT-IR spectrometer (PerkinElmer, Waltham, MA, USA), which operated within 4000–500 cm^−1^ to characterize the surface structure of the samples. Thermogravimetric analysis (TGA) (Evolution 2400, Setaram Instruments, Lyon, France) experiments were performed under an argon atmosphere between 30 and 550 °C at a heating rate of 10 °C/min. The mechanical performances of the sensor were characterized with a Shandong Liangong CMT-20 universal testing machine (Jinan, China). The electrical signals of the MCF strain sensor were recorded with a source meter (8845A, Fluke, Everett, WA, USA). The experiment was performed at room temperature (~19 °C) and ~40% relative humidity (RH).

## 3. Results and Discussions

**Figure 1:**
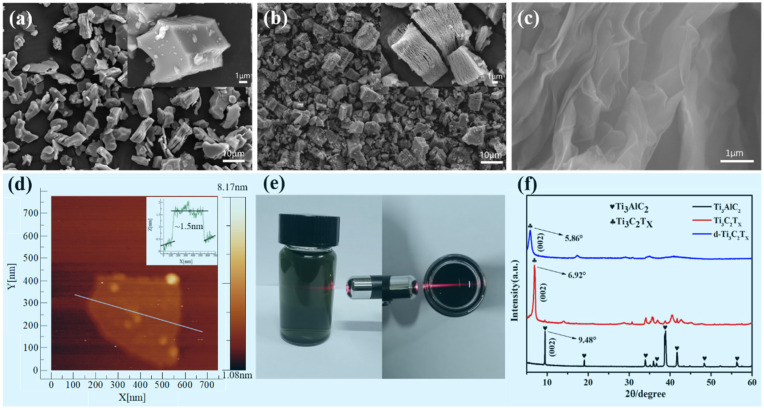
SEM images of (**a**) Ti_3_AlC; (**b**) unexfoliated Ti_3_C_2_T_x_; (**c**) exfoliated Ti_3_C_2_T_x_ nanosheets; (**d**) the atomic force microscopy (AFM) image of exfoliated Ti_3_C_2_T_x_ nanosheets; (**e**) the Tyndall effect in the Ti_3_C_2_T_x_ MXenes dispersion; and (**f**) the XRD patterns of Ti_3_AlC_2_, unexfoliated Ti_3_C_2_T_x_, and exfoliated Ti_3_C_2_T_x_.

SEM images of the Ti_3_AlC_2_ and MXenes Ti_3_C_2_T_x_ powder are shown in Figure 1a,b. The Ti_3_AlC_2_ raw material had the typical structure of a layered MAX phase (Figure 1a). After removing the Al atomic layers from the Ti_3_AlC_2_, the resulting MXene, Ti_3_C_2_T_x_, had an accordion-like multi-layered structure (Figure 1b). As seen in the SEM image of the d-Ti_3_C_2_T_x_ (as shown in Figure 1c), the MXene (Ti_3_C_2_T_x_) was delaminated into single- or few-layered d-Ti_3_C_2_T_x_ nanosheets. The thickness of the exfoliated MXene nanosheets was characterized by atomic force microscopy (AFM) (Figure 1d). AFM analysis indicated that the nanosheets had a thickness of about 1.5 nm (Figure 1d inset), confirming the d-Ti_3_C_2_T_x_ was composed of a single or a few layers of sheets. Moreover, the obtained dark green MXene nanosheet solution was a stable dispersion in water (Figure 1e) and exhibited an obvious Tyndall effect. The XRD patterns of the MAX phase, MXene, and exfoliated MXene nanosheets are shown in Figure 1f. Compared with the MAX phase, the characteristic peak of Ti_3_AlC_2_ disappeared in the MXene pattern and a new diffraction peak appeared at 6.9°, which belonged to the (002) orientation of MXene Ti_3_C_2_T_x_ [42]. After exfoliation, the (002) diffraction peak significantly shifted from 6.9° to a smaller angle of 5.86°, indicating that Ti_3_C_2_ was effectively delaminated into a single- or few-layered nanosheets.

**Figure 2:**
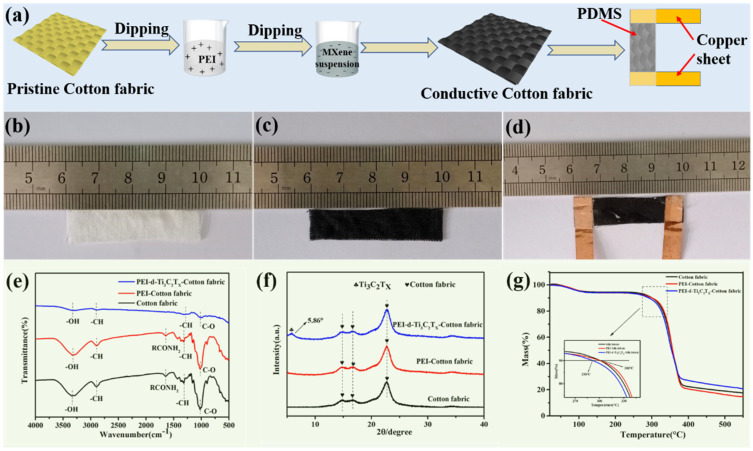
(**a–d**) Schematic illustration of MXene@Cotton Fabric (MCF) strain sensor fabrication process, (**e**) FTIR, (**f**) XRD and (**g**) TG-DSC data for the MCF strain sensors.

Figure 2a shows a schematic of the highly sensitive MXene/Cotton fabric strain sensor fabrication process. First, surface-modified cotton fabric was obtained by immersing clean fabric into a PEI solution for 24 h, followed by drying the fabric in an oven at 60 °C (Figure 2b). Then, the PEI-modified fabric was dipped into the MXene solution for 60 min. The MXene was adsorbed onto the fabric fiber due to the electrostatic attraction between the MXene and the positively charged PEI on the fabric (Figure 2c) during impregnation. Finally, the ends of the obtained MCF were coated with silver paste and attached to copper tape electrodes, and the material packaged within polydimethylsiloxane (PDMS) silicone rubber (Figure 2d).

Figure 2e shows the FTIR spectra of the fabric, fabric@PEI, and MCF, which gave insights into the functional groups present in the materials. As seen in Figure 2f, the broad peak around 3200~3500 cm^−1^ was attributed to the O–H/N–H stretching from the PEI and cotton cellulose of the fabric. The peak near 2910 cm^−1^ was assigned to the C–H stretching vibration band. The characteristic peaks at 1652, 1430~1310, and 1017 cm^−1^ were assigned to C=O stretching, C–H bending, and C–O stretching [43], respectively. These characteristic absorption peaks obviously weakened after the adsorption of the MXene. Therefore, it could be inferred that the fabric was wrapped in MXene nanosheets. To verify this, the sample also was characterized by XRD (Figure 2f). It can be found that in addition to the characteristic diffraction peak of the cotton fabric, a new peak appeared at 5.86° which was attributed to d-Ti_3_C_2_T_x_. In summary, the analytical results provided good evidence that the fabric surface was successfully capped with MXene d-Ti_3_C_2_T_x_ nanosheets. As can be seen from the TGA curves in Figure 2g, all samples completely evaporated at temperatures higher than 400 °C, and the MCF had the most ash residue. However, the thermal decomposition temperature was slightly lower due to the surface effect of nanomaterials. 

**Figure 3:**
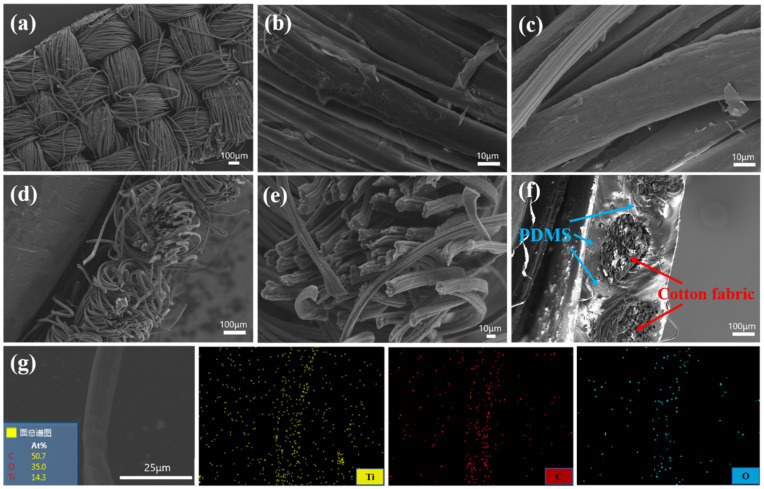
(**a**,**b**) SEM images of the surface of the clean cotton fabric at different magnifications; (**c**–**e**) SEM images of the surface of the conductive MCF from different angles; (**f**) SEM image of the fractured surface of the MXene/cotton fabric sensor after encapsulation in polydimethylsiloxane (PDMS); (**g**) SEM image of the MXene/cotton fabric fiber and the corresponding EDX elemental mapping (yellow: Ti, red: C, blue: O).

SEM images of the clean, conductive cotton fabric and the MCF strain sensor are shown in Figure 3. Figure 3a shows the morphology of the clean cotton fabric at different magnifications. The cotton fabric consisting of woven fiber bundles and the surface of fibers were relatively smooth. Figure 3c–e show the SEM images of the conductive MCF from different angles after dipping the fabric in the MXene suspension and drying. The smooth cotton fiber surface became rough after the flexible 2D MXene nanosheets decorated the fiber surface, and the assembled MXene nanosheets were observed on the cotton fibers. Hence, MXene decorated cotton fibers with a core–shell structure were obtained. Figure 3g is an SEM image of a MXene wrapped fiber and the corresponding elemental mapping. It was observed that Ti, C, and O were uniformly distributed on the cotton fiber surface, indicating that the fiber was tightly wrapped by a layer of MXene nanosheets. Figure 3f shows that the conductive cotton fibers were well encapsulated by the PDMS layers that play a protective and restrictive role for the inner conductive cotton fibers, and the fabric structure was maintained after the encapsulation process.

**Figure 4:**
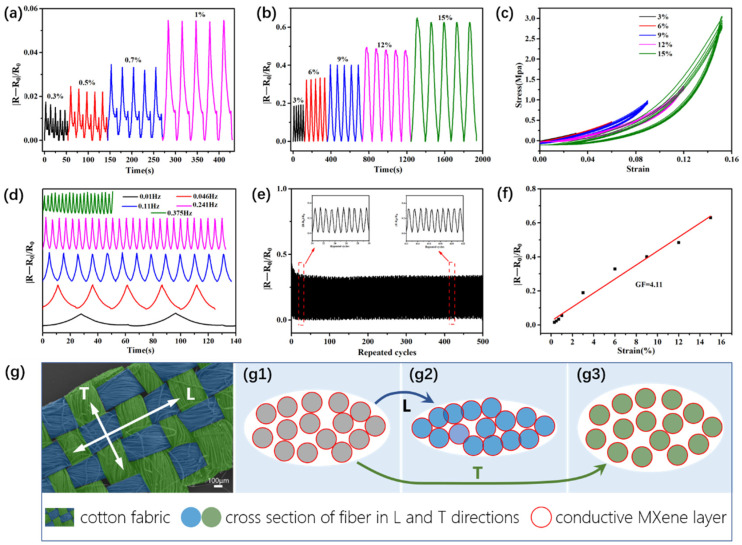
Strain sensing properties of the MCF strain sensors under (**a**) small (0.3, 0.5, 0.7 and 1.0%) and (**b**) large (3, 6, 9, 12, and 15%) strains; (**c**) stress–strain curves of the sensor; (**d**) relative resistance changes of the strain sensor at selected frequencies under a 9% strain; (**e**) cycling durability curve of the strain sensor under a 6% strain (stretching rate of 150 mm·min^−1^); (**f**) relative resistance variation–strain curve of the MCF strain sensors; and (**g**) the schematic diagram of the formation of a conductive pathway in the L and T directions.

Figure 4a,b show the resistance change rates (ΔR/R_0_) at different strains with repeated loading–unloading cycles under a tensile speed of 4 mm/min. During the stretching process, the tension led to a decrease in the yarn spacing, which resulted in the formation of conductive networks and a decrease in the resistance. The results showed that the greater the applied tension, the greater the measured change in the resistance rates. In addition, the corresponding ΔR/R_0_ values were almost constant after different loading–unloading cycles, which indicated the high cyclic stability of the MCF strain sensor material. Figure 4c shows the tensile stress–strain curves at different strains with repeated loading–unloading cycles. It could be found that the strain of the sensor returned to the initial value after five cycles under different strains. These results indicated that the MCF strain sensor exhibited excellent cyclic stability performance in mechanics. Figure 4d gives the ΔR/R_0_ of MCF strain sensor at different stretching frequencies under the same strain of 9%. These data suggested that the sensor also had a steady dynamic response to frequency changes from 0.01 to 0.375 Hz. The durability of the strain sensor under a tensile strain of 6% at a 150 mm/min strain rate is shown in Figure 4e. The strain sensor had a very stable signal output after 500 cycles of loading–unloading tests, showing excellent repeatability, which revealed that the different components in the MCF strain sensor were highly compatible, structurally stable, and are able to stretch and recover these properties during loading and unloading cycles. 

Figure 4g is the schematic diagram of the formation of the conductive pathways via the adsorption of conductive MXene on the surface of the fiber in the stretching direction (L direction) and vertical direction (T direction). Figure 4(g1) is the cross section of fiber bundle in an unstretched state, g2 is a stretched state in the L direction, and g3 is a stretched state in the T direction. The red circle represents the conductive MXene layer adsorbed on the surface of fiber, which will form a conductive network under certain conditions. Stretching the fabric in the L direction decreased the yarn spacing and made more contacts on the conductive MXene on the surface of parallel fiber, which led to the formation of a conductive network between the conductive strands of yarn (g2) in the L direction and the measured decrease in resistance. However, stretching the fabric in the L direction also led to an increase in the yarn spacing and the reconstruction of the conductive networks in the T direction, which will break down the conductive path and increase the resistance (g3). In both cases, the formation of conductive networks in the L direction played a major role in the resistance variation.

**Figure 5:**
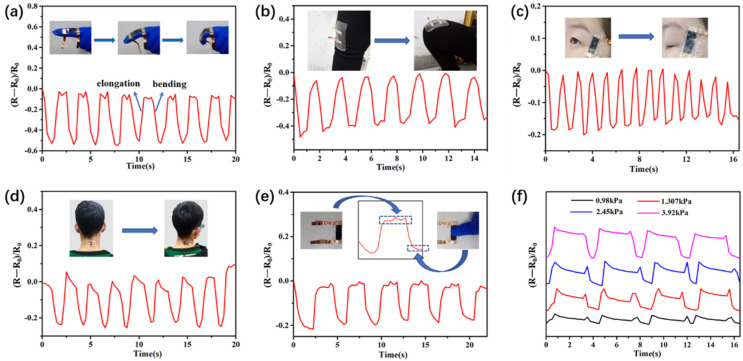
Responses of the MCF strain sensor to (**a**) finger bending; (**b**) squatting; (**c**) eye blinking; (**d**) neck twisting motions; and (**e**,**f**) applied pressure. Insets: Photographs of the MCF strain sensor attached onto a finger, knee, eye corner, and neck with the corresponding motions.

As mentioned above, the MCF strain sensor possessed high sensitivity under different strains. A series of tests were carried out to detect different human motions to verify the feasibility of using this strain sensor as a wearable electronic device. As a result, the ΔR/R_0_ value of MCF strain sensor increased and then returned to its initial state when the wearer tautologically bent their finger and leg (Figure 5a,b, respectively), achieving detection from small to large human body movements. Figure 5c demonstrates the detection of eye movements by attaching the MCF strain sensor to the corner of eye. The MCF sensor accurately recorded strain changes and showed regular variations in the resistance due to the repeated eye motions during blinking. When the sensor was attached to the neck, it showed a repeatable electrical signal instantaneously for twisting motions of the neck joint, as shown in Figure 5d. Furthermore, Figure 5e,f showed the stable response of the strain sensor under different pressures ranging from 0.98 to 3.92 kPa. Therefore, the MCF strain sensor may have potential application in wearable devices to monitor joint movements during human motion and health monitoring. 

## 4. Conclusions

We prepared a flexible, high-performance strain sensor based on cotton fabric and 2D MXene via a simple electrostatic self-assembly method. This flexible MCF piezoresistive pressure strain sensor had high sensitivity, a wide response range, and good stability. The MXene-coated cotton fabric acted as a flexible and simple strain sensor and produced a variety of signals according to body motions, such as the bending of a finger, squatting, blinking of an eye, and twisting of the neck. In addition, the flexible substrate, low active material content, and simple preparation process make the reported sensor more suitable for large-scale preparation and electronic skin applications. Moreover, this sensor is easily integrated into fabrics, which is significant for future applications in intelligent wearable devices.

## Data Availability

The data presented in this study are available within the article.

## References

[1] Wang J.L., Hassan M., Liu J.W., Yu S.H. (2018). Nanowire assemblies for flexible electronic devices: Recent advances and perspectives. Adv. Mater..

[2] Koo J.H., Kim D.C., Shim H.J., Kim T.H., Kim D.H. (2018). Flexible and stretchable smart display: Materials, fabrication, device design, and system integration. Adv. Funct. Mater..

[3] Lee T.D., Ebong A.U. (2017). A review of thin film solar cell technologies and challenges. Renew. Sustain. Energy Rev..

[4] Han S.T., Peng H., Sun Q., Venkatesh S., Chung K.S., Lau S.C., Zhou Y., Roy V.A.L. (2017). An overview of the development of flexible sensors. Adv. Mater..

[5] Kim H., Ahn J.H. (2017). Graphene for flexible and wearable device applications. Carbon.

[6] Gao W., Ota H., Kiriya D., Takei K., Javey A. (2019). Flexible electronics toward wearable sensing. Acc. Chem. Res..

[7] Wang X., Liu Z., Zhang T. (2017). Flexible sensing electronics for wearable/attachable health monitoring. Small.

[8] Ding Y., Xu T., Onyilagha O., Fong H., Zhu Z. (2019). Recent Advances in Flexible and Wearable Pressure Sensors Based on Piezoresistive 3D Monolithic Conductive Sponges. ACS Appl. Mater. Interfaces.

[9] Peng W., Han L., Huang H., Xuan X., Pan G., Wan L., Lu T., Xu M., Pan L. (2020). A direction-aware and ultrafast self-healing dual network hydrogel for flexible electronic skin strain sensor. J. Mater. Chem. A.

[10] Xia S., Song S., Jia F., Gao G. (2019). A flexible, adhesive and self-healable hydrogel-based wearable strain sensor for human motion and physiological signal monitoring. J. Mater. Chem. B.

[11] Li Q., Li K., Fan H., Hou C., Li Y., Zhang Q., Wang H. (2017). Reduced graphene oxide functionalized stretchable and multicolor electrothermal chromatic fibers. J. Mater. Chem. C.

[12] CHOI C., LEE J.M., KIM S.H., KIM S.J., Di J., Baughman R.H. (2016). Twistable and Stretchable Sandwich Structured Fiber for Wearable Sensors and Supercapacitors. Nano Lett..

[13] Dagdeviren C., Su Y., Joe P., Yona R., Liu Y., Kim Y., Huang Y., Damadoran A.R., Xia J., Martin L.W. (2014). Conformable amplified lead zirconate titanate sensors with enhanced piezoelectric response for cutaneous pressure monitoring. Nat. Commun..

[14] Zhang M., Wang C., Wang H., Jian M., Hao X., Zhang Y. (2017). Carbonized cotton fabric for high-performance wearable strain sensors. Adv. Funct. Mater..

[15] CHEN W., GUI X., LIANG B., Yang R., Zheng Y., Zhao C., Li X., Zhu H., Tang Z. (2017). Structural Engineering for High Sensitivity, Ultrathin PressureSensors Based on Wrinkled Graphene and Anodic Aluminum Oxide Membrane. ACS Appl. Interfaces.

[16] AN B., MA Y., LI W., Su M., Li F., Song Y. (2016). Three-dimensional multi-recognition flexible wearable sensor viagraphene aerogel printing. Chem. Commun..

[17] LI J., XU B. (2015). Novel highly sensitive and wearable pressure sensors from conductive three-dimensional fabric structures. Smart Mater. Struct..

[18] Seyedin S., Zhang P., Naebe M., Qin S., Chen J., Wang X., Razal J.M. (2019). Textile strain sensors: A review of the fabrication technologies, performance evaluation and applications. Mater. Horiz..

[19] Wang J., Lu C., Zhang K. (2020). Textile-Based Strain Sensor for Human Motion Detection. Energy Environ. Mater..

[20] Jian M., Wang C., Wang Q., Wang H., Xia K., Yin Z., Zhang M., Liang X., Zhang Y. (2017). Advanced carbon materials for flexible and wearable sensors. Sci. China Mater..

[21] Wang L., Chen Y., Lin L., Wang H., Huang X., Xue H., Gao J. (2019). Highly stretchable, anti-corrosive and wearable strain sensors based on the PDMS/CNTs decorated elastomer nanofiber composite. Chem. Eng. J..

[22] Zheng Y., Li Y., Zhou Y., Dai K., Zheng G., Zhang B., Liu C., Shen C. (2020). High-Performance Wearable Strain Sensor Based on Graphene/Cotton Fabric with High Durability and Low Detection Limit. ACS Appl. Mater. Interfaces.

[23] Kim S.R., Kim J.H., Park J.W. (2017). Wearable and transparent capacitive strain sensor with high sensitivity based on patterned Ag nanowire networks. ACS Appl. Mater. Interfaces.

[24] Yang H., Xue T., Li F., Liu W., Song Y. (2019). Graphene: Diversified flexible 2D material for wearable vital signs monitoring. Adv. Mater. Technol..

[25] Naguib M., Kurtoglu M., Presser V., Lu J., Niu J., Heon M., Hultman L., Gogotsi Y., Barsoum M.W. (2011). Two-dimensional nanocrystals produced by exfoliation of Ti_3_AlC_2_. Adv. Mater..

[26] Hasan M.M., Hossain M.M., Chowdhury H.K. (2021). Two-dimensional mxene-based flexible nanostructures for functional nanodevices: A review. J. Mater. Chem. A.

[27] Anasori B., Lukatskaya M.R., Gogotsi Y. (2017). 2D metal carbides and nitrides (MXenes) for energy storage. Nat. Rev. Mater..

[28] Morales-Garciía A., Calle-Vallejo F., Illas F. (2020). MXenes: New horizons in catalysis. ACS Catal..

[29] Tang H., Hu Q., Zheng M., Chi Y., Qin X., Pang H., Xu Q. (2018). MXene-2D layered electrode materials for energy storage. Prog. Nat. Sci. Mater. Int..

[30] Sun S., Liao C., Hafez A.M., Zhu H., Wu S. (2018). Two-dimensional MXenes for energy storage. Chem. Eng. J..

[31] Yun T., Kim H., Iqbal A., Cho Y.S., Lee G.S., Kim M., Kim S.J., Kim D., Gogotsi Y., Kim S.O. (2020). Electromagnetic shielding of monolayer MXene assemblies. Adv. Mater..

[32] Zhang H., Wang L., Chen Q., Li P., Zhou A., Cao X., Hu Q. (2016). Preparation, mechanical and anti-friction performance of MXene/polymer composites. Mater. Des..

[33] Wan Y.J., Li X.M., Zhu P.L., Sun R., Wong C., Liao W. (2020). Lightweight, flexible MXene/polymer film with simultaneously excellent mechanical property and high-performance electromagnetic interference shielding. Compos. Part A Appl. Sci. Manuf..

[34] Hwang S.K., Kang S.M., Rethinasabapathy M., Roh C., Huh Y.S. (2020). MXene: An emerging two-dimensional layered material for removal of radioactive pollutants. Chem. Eng. J..

[35] Jeon M., Jun B.M., Kim S., Jang M., Park C.M., Snyder S.A., Yoon Y. (2020). A review on MXene-based nanomaterials as adsorbents in aqueous solution. Chemosphere.

[36] Sinha A., Zhao H., Huang Y., Huang Y., Lu X., Chen J., Jain R. (2018). MXene: An emerging material for sensing and biosensing. Trac. Trends Anal. Chem..

[37] Guo Y., Zhong M., Fang Z., Wan P., Yu G. (2019). A wearable transient pressure sensor made with MXene nanosheets for sensitive broad-range human–machine interfacing. Nano Lett..

[38] Li H., Du Z. (2019). Preparation of a highly sensitive and stretchable strain sensor of MXene/silver nanocomposite-based yarn and wearable applications. ACS Appl. Mater. Interfaces.

[39] Li T., Chen L., Yang X., Chen X., Zhang Z., Zhao T., Li X., Zhang J. (2019). A flexible pressure sensor based on an MXene-textile network structure. J. Mater. Chem. C.

[40] Liu R., Li J., Li M., Zhang Q., Shi G., Li Y., Hou C., Wang H. (2020). MXene-coated air-permeable pressure-sensing fabric for smart wear. ACS Appl. Mater. Interfaces.

[41] Li L., Zhou A., Xu L., Li Z., Wang L. (2013). Synthesis of high pure Ti_3_AlC_2_ and Ti_2_AlC powders from TiH_2_ powders as Ti source by tube furnace. J. Wuhan Univ. Technol. Sci. Ed..

[42] Liu F., Zhou A., Chen J., Jia J., Zhou W., Wang L., Hu Q. (2017). Preparation of Ti_3_C_2_ and Ti_2_C MXenes by fluoride salts etching and methane adsorptive properties. Appl. Surf. Sci..

[43] Cai G., Xu Z., Yang M., Tang B., Wang X. (2017). Functionalization of cotton fabrics through thermal reduction of graphene oxide. Appl. Surf. Sci..

